# *PAX7*, *PAX9* and *RYK* Expression in Cleft Affected Tissue

**DOI:** 10.3390/medicina57101075

**Published:** 2021-10-08

**Authors:** Mārtiņš Vaivads, Ilze Akota, Māra Pilmane

**Affiliations:** 1 Department of Morphology, Institute of Anatomy and Anthropology, Riga Stradins University, Kronvalda Boulevard 9, LV-1010 Riga, Latvia; Mara.Pilmane@rsu.lv; 2 Department of Oral and Maxillofacial Surgery, Riga Stradins University, 16 Dzirciema Street, LV-1007 Riga, Latvia; Ilze.Akota@rsu.lv; 3 Cleft Lip and Palate Centre, Institute of Stomatology, Riga Stradins University, 20 Dzirciema Street, LV-1007 Riga, Latvia

**Keywords:** cleft lip, cleft palate, *PAX7*, *PAX9*, *RYK*

## Abstract

*Background and Objectives*: Cleft lip with or without cleft palate is one of the most common types of congenital malformations. Transcription factors paired box 7 and 9 (*PAX7*, *PAX9*) and receptor-like tyrosine kinase (*RYK*) have been previously associated with the formation of orofacial clefts but their exact possible involvement and interactions in the tissue of specific cleft types remains uncertain. There is a limited number of morphological studies analyzing these specific factors in cleft affected tissue due to ethical aspects and the limited amount of available tissue material. This study analyses the presence of *PAX7*, *PAX9*, and *RYK* immunopositive structures within different cleft affected tissue to assess their possible involvement in cleft morphopathogenesis. *Materials and Methods*: Cleft affected tissue was collected from non-syndromic orofacial cleft patients during cleft correcting surgery (36 patients with unilateral cleft lip, 13 patients with bilateral cleft lip, 26 patients with isolated cleft palate). Control group oral cavity tissue was obtained from 7 patients without cleft lip and palate. To evaluate the number of immunopositive structures in the cleft affected tissue and the control group, a semiquantitative counting method was used. Non-parametric statistical methods (Kruskal–Wallis H test, Mann–Whitney U test, and Spearman’s rank correlation) were used. *Results*: Statistically significant differences for the number of *PAX7*, *PAX9*, and *RYK*-positive cells were notified between the controls and the patient groups. Multiple statistically significant correlations between the factors were found in each cleft affected tissue group. *Conclusions*: *PAX7*, *PAX9*, and *RYK* have a variable involvement and interaction in postnatal morphopathogenesis of orofacial clefts. *PAX7* is more associated with the formation of unilateral cleft lip, while *PAX9* relates more towards the isolated cleft palate. The stable presence of *RYK* in all cleft types indicates its possible participation in different facial cleft formations.

## 1. Introduction

Cleft lip and palate are relatively common congenital malformations, and they cause excessive functional disabilities in affected children and increase socioeconomic burden and suffering within affected individuals and their families. There are multiple possible propositions for the pathogenesis of cleft lip and palate, but the etiology of orofacial clefts is mainly understood as multifactorial in nature with the involvement of both environmental factors and individual genetic factors [1,2]. The complicated interactions between the surrounding external environmental factors during pregnancy and multiple genes involved in craniofacial region development can initiate and impact the formation of different orofacial clefts. Multiple different cleft candidate gene interactions and mutations have been associated with the development of craniofacial clefts, for example, the involvement of paired box (PAX) genes such as *PAX7* and *PAX9* in some orofacial cleft cases [2,3]. Improved understanding of the presence and interactions of specific cleft candidate genes and their products within cleft affected tissue could help acknowledge some of the possible cleft pathogenesis variations.

Multiple classification systems have been used to organize the different types of orofacial clefts, and the classification of clefts can be based on morphology, anatomy, and etiology [1]. Cleft lip and palate can be classified by phenotype as isolated cleft lip without cleft palate (including both unilateral and bilateral cleft lip), cleft lip with cleft palate, and isolated cleft palate [2]. The incidence of orofacial clefts varies and depends on the population and geographic region. Cleft lip and palate, on average, affects approximately 1 per 700 live births globally [4]. The formation of non-syndromic orofacial clefts has been associated with multiple genetic factors, which affect the development of the orofacial region. In this study, the presence of *PAX7, PAX9*, and *RYK* within cleft affected tissue was analyzed.

Paired box 7 (*PAX7*) is a transcription factor that is involved with the formation of the craniofacial region by regulating cranial neural crest cell development and differentiation [5]. Dysfunction and impairment of *PAX7* have been associated with the formation of craniofacial clefts both within mice and in the human population [5,6]. *PAX7* has been mainly described as an essential factor for myogenesis by maintaining a population of myosatellite cells and regulating correct skeletal muscle tissue development [7,8] but *PAX7* is also involved with the formation of the craniofacial region by regulating correct tissue morphogenesis, neural crest cell survival, patterning, and correct specification of the frontonasal structures [5].

Paired box 9 (*PAX9*) is an RNA polymerase II transcription factor that regulates protein expression during the development of the orofacial region and is involved in tooth development [9]. *PAX9* is necessary for the formation of the small ribosome subunit and depletion or dysfunction of *PAX9* leads to the disruption in cranial neural crest cell development, leading to the formation of craniofacial anomalies, including orofacial clefts [9,10]. *PAX9* also provides an important regulatory role during mesenchyme–epithelial interactions within the palatogenesis process by regulating Bmp4, Fgf10, Osr2, and Shh pathways in the developing palatal tissue during embryogenesis [11]. *PAX9* gene mutations have been associated with oligodontia, tooth agenesis, and the development of cleft palate [11,12].

Receptor-like tyrosine kinase (*RYK*) has been previously proposed as a cleft candidate gene [13,14]. *RYK* belongs to a group of conserved transmembrane molecules that have an extracellular domain that resemble the Wnt inhibitory factor (WIF) protein [15]. *RYK* has a tyrosine kinase motif within the molecular structure but data from a sequential analysis has shown that *RYK* is not an active tyrosine kinase enzyme and may function as a coreceptor within the Wnt signaling pathway [15]. *RYK* is involved in processes of neurogenesis, ectoderm development and differentiation, and craniofacial region formation during embryogenesis [16,17]. In a study analyzing mice with the null allele of *RYK*, a complete cleft of the secondary palate and a specific craniofacial appearance was notified [18].

This study analyses the presence of *PAX7, PAX9*, and *RYK* immunopositive structures within different cleft affected tissues to reveal their possible involvement in cleft morphopathogenesis.

## 2. Materials and Methods

The study was conducted in accordance with the 1964 Declaration of Helsinki. All tissue samples used for the study were taken from patients with a voluntary agreement from the parents of patients from each patient group and the parents of controls to allow the donation of the tissue samples for scientific research. Patient and control group tissue samples were acquired from the Cleft Lip and Palate Centre of the Institute of Stomatology of Riga Stradins University (RSU) and the analysis and study was performed in the Department of Morphology of RSU. The Ethics committee of RSU provided the approval of the study protocol (22.05.2003.; Nr.6-1/10/11, 24.09.2020).

The study groups were divided based on the cleft type (unilateral cleft lip, bilateral cleft lip, isolated cleft palate). The soft cleft tissues with the oral cavity epithelium and the underlying connective tissue were taken during cleft surgery. The inclusion criteria for the patient groups were the following: diagnosis and surgery of non-syndromic unilateral cleft lip, bilateral cleft lip, and isolated cleft palate, respectively, patient age before primary dentition (age 3–18 months), no periodontal disease detected, or no other pathology which would impede the patient from receiving cleft lip and palate reparative surgery.

For the unilateral cleft lip group, 36 patients participated in the study (20 boys and 16 girls) aged 3–8 months. For the bilateral cleft lip group, 13 patients participated in the study (10 boys and 3 girls) aged 4–16 months. For the isolated cleft palate group, 26 patients participated in the study (18 boys and 8 girls) aged 4–14 months.

Control group oral cavity tissue was taken from 7 patients who received labial frenectomy due to the surgical correction of hypertrophic upper lip frenulum. The structure of the control group was composed of four boys and three girls (8–11 years old). The control group inclusion criteria were the following: patients with the diagnosis of hypertrophic upper lip frenulum, no inflammation and no other pathological process detected in the tissue sample, no craniofacial clefts in anamnesis or in family history.

Due to the very limited amount of control group tissue material, *PAX7* immunoreactivity could be evaluated from only 5 control group patients. *PAX9* and *RYK* immunoreactivity could be evaluated from all 7 control group patients.

Standard biotin and streptavidin immunohistochemical method was performed for the detection of *PAX7*, *PAX9*, and *RYK* [19]. The tissue samples were fixed in 2% formaldehyde and 0.2% picric acid in 0.1 M phosphate buffer (pH 7.2). The washing procedure was performed in phosphate-buffered saline (PBS) fluid containing 10% saccharose for 12 h. The embedding procedure was performed in paraffin and cutting was performed into 6–7 μm thick sections. Later, deparaffinization was carried out and further slide staining was performed with the biotin-streptavidin immunohistochemical method for detection of the presence of specific proteins within the tissue with antibodies for *PAX7* (ab55494, 1:100, Abcam, Cambridge, UK), *PAX9* (orb11242, 1:100, Biorbyt Ltd., Cambridge, UK), and *RYK* (orb38371, 1:100, Biorbyt Ltd., Cambridge, UK).

The visual illustration of slides was provided by Leica DC 300F digital camera (Leica Microsystems GmbH, Wetzlar, Germany). Further processing of images and image analysis was performed with Image-Pro Plus software (Media Cybernetics, Inc., Rockville, MD, USA).

A semi-quantitative counting method was used to record and provide a non-parametric evaluation of the relative frequency of immunopositive cells by using the immunohistochemical method [20]. The relative frequency of positively stained cells was analyzed with light microscopy in five visual fields of each section by two independent researchers. No positive structures or cells were labeled as 0, a rare occurrence of positive structures was labeled as 0/+, a few positive structures were labeled as +, a few to moderate number of positive structures: +/++, moderate number of positive structures: ++, moderate to numerous number of positive cells: ++/+++, numerous number of positive cells: +++, numerous to abundant number of positive structures: +++/++++, and an abundance of positive cells in the visual field was labeled as ++++.

Analysis of data was performed by using both analytical and descriptive statistical methods. The count of *PAX7, PAX9*, and *RYK* positive cells per each visual field, median value, and interquartile range calculation was performed for further evaluation using Spearman’s rank correlation analysis. Spearman’s rank correlation coefficient’s Spearman’s rho value (r_s_) was interpreted as the following values: r_s_ = 0.0–0.2, a very weak correlation; r_s_ = 0.2–0.4, a weak correlation; r_s_ = 0.4–0.6, a moderate correlation; r_s_ = 0.6–0.8, a strong correlation; r_s_ = 0.8–1.0, a very strong correlation. The semi-quantitative count of immunoreactive structures is shown as median values. Statistical significance was calculated with the Kruskal–Wallis H test and Mann–Whitney U test between each group. The statistical analysis of data was provided with the statistics program SPSS Statistics (version 25.0, IBM Company, Chicago, IL, USA). A *p*-value of <0.05 was considered statistically significant for all statistical calculations.

## 3. Results

### 3.1. Routine Hematoxylin and Eosin-Stained Slide Evaluation

**Figure 1:**
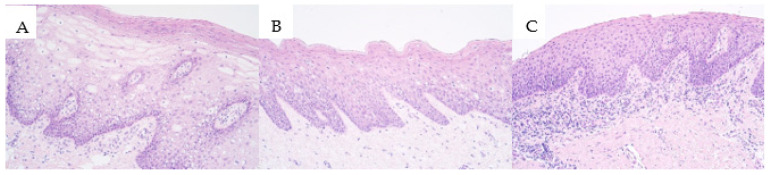
Micrographs of cleft affected tissue stained with hematoxylin and eosin (H&E). (**A**) Unilateral cleft lip affected tissue with visible vacuolization within the oral epithelium, hematoxylin and eosin (H&E), 200×. (**B**) Bilateral cleft lip affected tissue with some patchy vacuolization within the epithelium, H&E, 200×. (**C**) Isolated cleft palate affected tissue with visible subepithelial inflammation, H&E, 200×.

Hematoxylin and eosin-stained slides for all three patient groups were prepared to notify the presence of the surface epithelium and the underlying connective tissue. In all slides of the patient groups, stratified squamous epithelium with underlying connective tissue was found. The tissue fragments in all three patient groups were mainly similar to a relatively normal oral cavity and lip tissue (without inflammation, without fibrotic changes, and without vacuolization of the epithelium) with some slight variations. These variations include the presence of minimal subepithelial inflammation with infiltration of inflammatory cells (more visible in the isolated cleft palate group with seven individuals having minor subepithelial inflammation when compared to five individuals in the unilateral cleft lip group and two individuals within the bilateral cleft lip affected tissue group). Relatively minor vacuolization (a few to moderate number of epitheliocytes) within the surface epithelium was notified in four individuals within the unilateral cleft lip group and in one individual within the bilateral cleft lip group, but epithelial vacuolization was not notified in the isolated cleft palate tissue group. In some cleft affected tissue slides, patchy vacuolization of the oral cavity epithelium was visible in epithelial cells (Figure 1A,B). A patchy proliferation of the basal cells of the oral cavity epithelium was noticed in some slides. In some isolated cleft palate slides, the presence of subepithelial inflammation with fibrotic changes in the connective tissue was visible (Figure 1C).

### 3.2. PAX7 Immunohistochemical Evaluation

The number of factor positive cells found in the different cleft affected tissue groups and the controls was quite variable.

**Figure 2:**
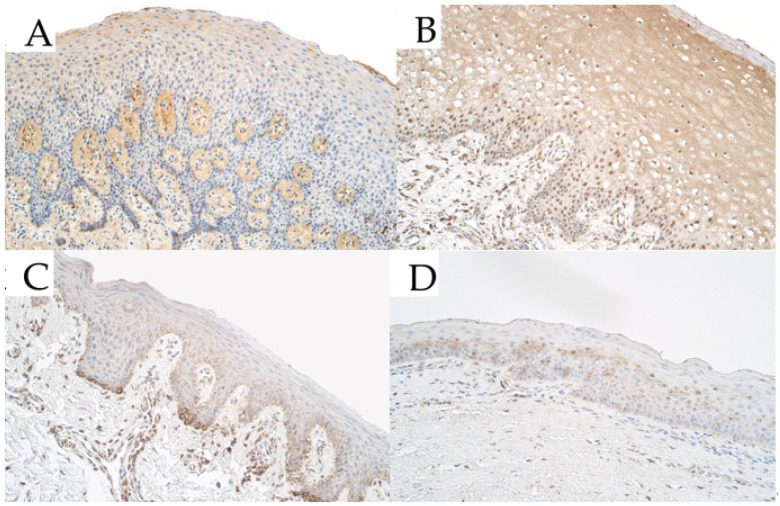
Immunoreactive structures for paired box 7 (*PAX7*) in cleft affected tissue. (**A**) Control group tissue with moderate *PAX7*-positive epitheliocytes in the epithelium and moderate to numerous *PAX7*-positive structures in the connective tissue, *PAX7* immunohistochemistry (IMH), 200×. (**B**) Unilateral cleft lip affected tissue with numerous *PAX7*-positive epitheliocytes in the epithelium and moderate to numerous *PAX7*-positive cells in the connective tissue, *PAX7* IMH, 200×. (**C**) Bilateral cleft lip affected tissue with a moderate number of weakly stained *PAX7*-positive epitheliocytes in the epithelium and few to moderate *PAX7*-positive cells in the connective tissue, *PAX7* IMH, 200×. (**D**) Isolated cleft palate affected tissue with a few to moderate number of *PAX7*-positive epitheliocytes in the epithelium and a few *PAX7*-positive structures in the connective tissue, *PAX7* IMH, 200×.

Within the control group, the median number of *PAX7*-positive epitheliocytes in the epithelium was few to moderate (+/++) and it ranged from a few (+) to moderate (++) number of *PAX7*-positive cells. In the connective tissue of the control group, the median number of *PAX7*-positive connective tissue cells was few to moderate (+/++) and ranged from a few (+) to moderate to numerous (++/+++) *PAX7*-positive cells (Figure 2A).

For *PAX7* within unilateral cleft lip affected tissue, the median number of *PAX7* containing epitheliocytes was moderate to numerous (++/+++) and the number of factor-positive cells ranged from few to moderate (+/++) to numerous to abundant (+++/++++). Within the connective tissue of the unilateral cleft lip group, the median number of *PAX7*-containing positive cells was numerous (+++) and ranged from a few to moderate (+/++) to abundant (++++) positive cells which were mainly macrophages, fibroblasts, and endothelial cells (Figure 2B).

For *PAX7* in bilateral cleft lip affected tissue, the median number of *PAX7*-positive epiheliocytes was moderate (++) and the number of factor-positive epitheliocytes ranged from a few (+) to numerous (+++) *PAX7*-positive cells. For *PAX7* in bilateral cleft lip affected connective tissue, the median number of factor-positive cells (*PAX7* was mainly found in macrophages and also in some fibroblasts) was few to moderate (+/++) and ranged from a few (+) to moderate to numerous (++/+++) within the bilateral cleft lip patient group (Figure 2C).

In isolated cleft palate affected tissue, the median number of *PAX7*-containing epitheliocytes was a few to moderate (+/++) within the epithelium and ranged from barely detectable *PAX7*-positive cells (0/+) to numerous to abundant (+++/++++). The median number of *PAX7*-positive cells in isolated cleft palate affected tissue was moderate to numerous (++/+++) and the number of *PAX7*-positive cells (fibroblasts, macrophages, and endothelial cells) in connective tissue ranged from a few (+) to numerous to abundant (+++/++++) (Figure 2D).

The use of the Kruskal–Wallis H test notified that a statistically significant difference was found in the number of *PAX7*-positive structures in the epithelium between the controls, unilateral cleft lip, bilateral cleft lip, and isolated cleft palate groups (H = 25.804, df = 3, *p* < 0.001). The Kruskal–Wallis H test also indicated a statistically significant difference in the number of *PAX7*-positive structures in the connective tissue between the controls, unilateral cleft lip, bilateral cleft lip, and isolated cleft palate groups (H = 28.955, df = 3, *p* < 0.001).

The Mann–Whitney U test notified a statistically significant difference in the number of *PAX7*-positive epitheliocytes in the epithelium between the control group and unilateral cleft lip affected tissue group (U = 5.5, *p* = 0.001). A statistically significant difference was also seen for the number of *PAX7*-containing cells within the connective tissue between the control group and the unilateral cleft lip affected tissue group (U = 15.5, *p* = 0.002).

The Mann–Whitney U test notified that no statistically significant difference was detected in the number of *PAX7*-positive epitheliocytes in the epithelium between the bilateral cleft lip affected tissue group (U = 18.5, *p* = 0.154). The Mann–Whitney U test also indicated that was no statistically significant difference present in the number of *PAX7*-positive cells within the connective tissue between the controls and the bilateral cleft lip affected tissue group (U = 32.0, *p* = 0.959).

The Mann–Whitney U test indicated that no statistically significant difference was notified for the number of *PAX7*-containing epitheliocytes in the epithelium between the control group and isolated cleft palate group (U = 46.0, *p* = 0.481). No statistically significant difference was found in the number of *PAX7*-positive cells in the connective tissue between the controls and the isolated cleft palate affected tissue (U = 34.5, *p* = 0.091).

### 3.3. PAX9 Immunohistochemical Evaluation

**Figure 3:**
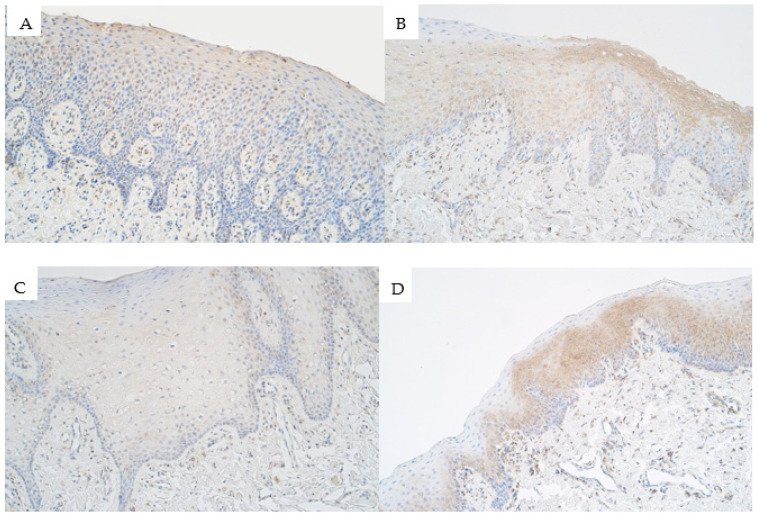
Immunoreactive structures for paired box 9 (*PAX9*) in cleft affected tissue. (**A**) Control group tissue with a moderate number of *PAX9*-containing epitheliocytes in the surface epithelium and barely detectable *PAX9*-positive cells in the connective tissue, *PAX9* IMH, 200×. (**B**) Unilateral cleft lip affected tissue with moderate number of *PAX9* epitheliocytes in the patient epithelium and a few *PAX9*-containing structures in the connective tissue, *PAX9* IMH, 200×. (**C**) Bilateral cleft lip affected tissue with a few *PAX9*-positive structures in the surface epithelium and a few *PAX9*-containing cells in the connective tissue, *PAX9* IMH, 200×. (**D**) Isolated cleft palate affected tissue with a moderate number of *PAX9*-positive epitheliocytes in the surface epithelium and a few *PAX9*-positive structures in the connective tissue, *PAX9* IMH, 200×.

Within the control group, the median number of *PAX9*-containing epithaliocytes in the epithelium was moderate (++) and it ranged from moderate (++) to moderate to numerous (++/+++) *PAX9*-positive cells. Within the connective tissue of the control group, the median number of *PAX9*-positive structures was 0 (no *PAX9*-positive cells) and it ranged from no *PAX9*-positive cells (0) to barely detectable (0/+) (Figure 3A).

The median number of *PAX9*-positive epitheliocytes in the epithelium of unilateral cleft lip affected tissue was few to moderate (+/++) and ranged from no *PAX9*-containing cells (0) to numerous (+++) *PAX9*-positive cells within the unilateral cleft lip patient group. The median number of *PAX9*-positive connective tissue cells such as fibroblasts, macrophages, and endothelial cells within the connective tissue of the unilateral cleft lip patient group was moderate (++) and ranged from no positive cells (0) to numerous (+++) positive cells (Figure 3B).

The median number of *PAX9*-positive cells within the epithelium of bilateral cleft lip patient group tissue was a few (+) positive cells and ranged from no positive epitheliocytes (0) to moderate to numerous (++/+++) positive epitheliocytes. Within the connective tissue of the bilateral cleft lip patient group tissue, the median number of *PAX9*-positive cells was a barely detectable (0/+) number of *PAX9*-containing cells and the values ranged from no positive structures (0) to a moderate number (++) of *PAX9*-containing cells (Figure 3C).

The median number of *PAX9*-positive epitheliocytes in the surface epithelium of isolated cleft palate affected tissue was a barely detectable number of positive cells (0/+) and ranged from no positive cells (0) to moderate (++) number of *PAX9*-containing cells. The median number of *PAX9*-positive cells within the connective tissue of the isolated cleft palate patient group was a few (+) immunopositive cells (mainly endothelial cells and some macrophages) and had a range from no positive cells (0) to moderate to numerous (++/+++) in some patients (Figure 3D).

The Kruskal–Wallis H test notified a statistically significant difference for the number of *PAX9*-positive structures in the epithelium between the control group, unilateral cleft lip group, bilateral cleft lip group, and isolated cleft palate group (H = 28.308, df = 3, *p* < 0.001). Kruskal–Wallis H test also indicated that there was a statistically significant difference for the number of *PAX9*-positive structures in the connective tissue between the control group, unilateral cleft lip group, bilateral cleft lip group, and isolated cleft palate group (H = 33.917, df = 3, *p* < 0.001).

The Mann–Whitney U test notified that no statistically significant difference was found for the number of *PAX9*-containing epitheliocytes within the epithelium between the control group and the unilateral cleft lip affected tissue group (U = 78.0, *p* = 0.107). There was a statistically significant difference in the number of *PAX9*-containing cells in the connective tissue between the control group and the unilateral cleft lip affected tissue group (U = 9.5, *p* < 0.001).

The Mann–Whitney U test indicated a statistically significant difference for the number of *PAX9*-positive epitheliocytes in the surface epithelium between the control group and the bilateral cleft lip affected tissue group (U = 13.5, *p* = 0.012). Mann–Whitney U test also indicated no statistically significant difference for the number of *PAX9*-positive cells within the connective tissue between the control group and the bilateral cleft lip affected tissue group (U = 29.0, *p* = 0.155).

The Mann–Whitney U test notified a statistically significant difference for the number of *PAX9*-positive epitheliocytes in the surface epithelium between the control group and the isolated cleft palate affected tissue group (U = 2.5, *p* < 0.001). Mann–Whitney U test indicated a statistically significant difference for the number of *PAX9*-positive cells within the connective tissue between the control group and the isolated cleft palate affected tissue group (U = 18.5, *p* = 0.001).

### 3.4. RYK Immunohistochemical Evaluation

**Figure 4:**
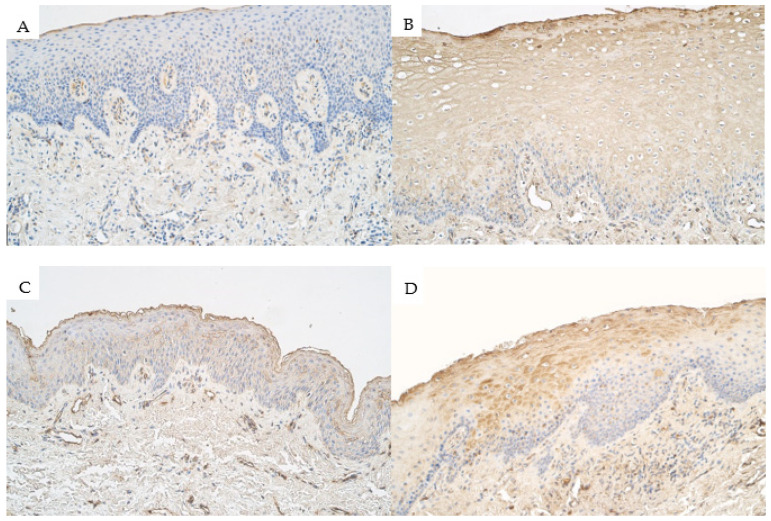
Immunoreactive structures for receptor-like tyrosine kinase (*RYK*) in cleft affected tissue. (**A**) Control group tissue with a few *RYK*-containing epitheliocytes in the epithelium and a few *RYK*-containing cells in the connective tissue, *RYK* IMH, 200×. (**B**) Unilateral cleft lip affected tissue with numerous *RYK*-positive epitheliocytes in the epithelium and moderate to numerous *RYK*-containing cells in the connective tissue, *RYK* IMH, 200×. (**C**) Bilateral cleft lip affected tissue with a moderate number of *RYK*-positive structures in the epithelium and moderate to numerous *RYK*-positive structures in the connective tissue, and also in the endothelium, *RYK* IMH, 200×. (**D**) Isolated cleft palate affected tissue with moderate to numerous *RYK*-containing epitheliocytes within the epithelium and a moderate number *RYK*-containing cells in the connective tissue, *RYK* IMH, 200×.

Within the control group, the median number of *RYK*-positive epitheliocytes in the epithelium was a barely detectable (0/+) number of immunopositive cells and it ranged from no positive cells (0) to moderate (++) number of *RYK*-containing cells. Within the connective tissue of the control group, the median number of *RYK*-containing connective tissue cells was a few (+) positive cells and it ranged from no *RYK*-containing cells (0) to a moderate (++) number of *RYK*-positive cells (Figure 4A).

For *RYK*, the median number of immunopositive cells within the epithelium of the unilateral cleft lip patient group was moderate to numerous (++/+++) and ranged from few to moderate (+/++) number to numerous to abundant (+++/++++) number of *RYK*-positive cells. In the connective tissue of the unilateral cleft lip patient group, the median number of *RYK*-containing cells (mainly endothelial cells and macrophages) was moderate to numerous (++/+++) and ranged from a few (+) positive cells to numerous (+++) (Figure 4B).

The median number of *RYK*-containing epitheliocytes in the epithelium of the bilateral cleft lip patient group was moderate to numerous (++/+++) and ranged from few to moderate (+/++) to numerous (+++). The median number of *RYK*-containing cells (mostly endothelial cells and some macrophages) within the connective tissue of the bilateral cleft lip patient group was moderate to numerous (++/+++) and ranged from few to moderate (+/++) to numerous (+++) (Figure 4C).

The median number of *RYK*-containing epitheliocytes in the epithelium of isolated cleft palate patient group tissue was moderate to numerous (++/+++) and ranged from few to moderate (+/++) to abundant (++++). The median number of *RYK*-containing connective tissue cells (mostly macrophages, fibroblasts, and endothelial cells) in the connective tissue of the isolated cleft palate patient group was moderate to numerous (++/+++) and ranged from few to moderate (+/++) to numerous to abundant (+++/++++) in number (Figure 4D).

The Kruskal–Wallis H test indicated a statistically significant difference for the number of *RYK*-positive structures in the epithelium between the controls, unilateral cleft lip group, bilateral cleft lip group, and isolated cleft palate group (H = 22.868, df = 3, *p* < 0.001). Kruskal–Wallis H test also notified that no statistically significant difference was found in the number of *RYK*-positive structures in the connective between the control group, unilateral cleft lip patient group, bilateral cleft lip patient group, and isolated cleft palate patient group (H = 18.307, df = 3, *p* < 0.001).

The Mann–Whitney U test indicated a statistically significant difference for the number of *RYK*-containing epitheliocytes in the epithelium (U = 5.5, *p* < 0.001) and the connective tissue (U = 11.0, *p* < 0.001) between the control group and the unilateral cleft lip affected tissue group.

The Mann–Whitney U test indicated a statistically significant difference for the number of *RYK*-positive epitheliocytes in the epithelium between the control group and the bilateral cleft lip affected tissue group (U = 3.0, *p* = 0.001). A statistically significant difference was also found in the number of *RYK*-containing epitheliocytes in the connective tissue between the control group and the isolated cleft palate affected tissue (U = 3.0, *p* = 0.001).

The Mann–Whitney U test calculation notified statistically significant differences between the control group and the isolated cleft palate affected tissue group in the number of *RYK*-containing cells within the epithelium (U = 10.0, *p* < 0.001) and in the connective tissue (U = 5.0, *p* < 0.001).

The semiquantitative evaluation of *PAX7*, *PAX9*, and *RYK* immunoreactivity is summarized in Table 1.

### 3.5. Correlations

Spearman’s rank correlation coefficient correlation calculation showed statistically significant correlations between the number of immunopositive structures for *PAX7*, *PAX9*, and *RYK* within the epithelium and the connective tissue within each type of cleft tissue analyzed in the study (unilateral cleft lip patient group, bilateral cleft lip patient group, and isolated cleft palate patient group). No statistically significant correlations were found within the control group tissue.

#### 3.5.1. Correlations in Unilateral Cleft Lip Affected Tissue

In unilateral cleft lip affected tissue, a statistically significant strong correlation (Spearman’s rho (r_s)_ = 0.6–0.8) was notified between the number of *PAX9*-containing epitheliocytes in the epithelium and the number of *PAX9*-containing cells within the connective tissue (r_s_ = 0.618, *p* < 0.001). In the unilateral cleft lip patient group, moderate correlations (r_s_ = 0.4–0.6) were detected between the number of *PAX7*-containing epitheliocytes in the epithelium and the number of *PAX7*-containing cells in the connective tissue (r_s_ = 0.572, *p* < 0.001), between the number of *RYK*-positive cells in the epithelium and the number of *RYK*-containing cells in the connective tissue (r_s_ = 0.562, *p* < 0.001), between the number of *PAX7*-positive structures in the connective tissue and the number of *PAX9*-positive structures in the connective tissue (r_s_ = 0.531, *p* = 0.001), between the number of *RYK*-containing epitheliocytes in the epithelium and the number of *PAX9*-containing cells in the connective tissue (r_s_ = 0.430, *p* = 0.009), between the number of *RYK*-containing epitheliocytes in the epithelium and the number of *PAX7*-containing epitheliocytes in the epithelium (r_s_ = 0.407, *p* = 0.014), between the number of *RYK*-containing epitheliocytes within the epithelium and the number of *PAX9*-containing epitheliocytes within the epithelium (r_s_ = 0.400, *p* = 0.016). A weak correlation (r_s_ = 0.2–0.4) in the unilateral cleft lip patient group was notified between the number of *RYK*-containing structures in the connective tissue and the number of *PAX9*-containing structures in the connective tissue (r_s_ = 0.354, *p* = 0.034). The correlations between the factors in unilateral cleft lip affected tissue are summarized in Table 2.

#### 3.5.2. Correlations in Bilateral Cleft Lip Affected Tissue

In the bilateral cleft lip affected tissue, very strong statistically significant correlations (r_s_ = 0.8–1.0) were seen between the number of *PAX9*-containing epitheliocytes in the epithelium and the number of *PAX9*-containing connective tissue cells (r_s_ = 0.882, *p* < 0.001), between the number of *PAX7*-containing epitheliocytes in the epithelium and the number of *RYK*-containing cells in the epithelium (r_s_ = 0.869, *p* < 0.001). The correlations between the factors in bilateral cleft lip affected tissue can be found in Table 3.

#### 3.5.3. Correlations in Isolated Cleft Palate Affected Tissue

In the isolated cleft palate affected tissue, strong correlations (r_s_ = 0.6–0.8) were detected between the number of *PAX7*-containing epitheliocytes within the epithelium and the number of *RYK*-containing cells in the epithelium (r_s_ = 0.685, *p* < 0.001), between the number of *PAX9*-containing epitheliocytes in the epithelium and the number of *PAX9*-positive structures within the connective tissue (r_s_ = 0.674, *p* < 0.001).

Multiple statistically significant moderate correlations (r_s_ = 0.4–0.6) were discovered between the number of *PAX7*, *PAX9*, and *RYK*-positive structures within the epithelium and connective tissue found in the isolated cleft palate affected tissue. Moderate correlations were found between the number of *PAX9*-containing epitheliocytes in the epithelium and the number of *PAX7*-containing connective tissue cells (r_s_ = 0.549, *p* = 0.007), between the number of *PAX9*-containing surface epitheliocytes and the number of *PAX7*-containing connective tissue cells (r_s_ = 0.563, *p* = 0.003), between the number of *PAX9*-containing surface epitheliocytes and the number of *RYK*-containing epitheliocytes in the epithelium (r_s_ = 0.524, *p* = 0.010), between the number of *PAX9*-positive connective tissue cells and the number of *PAX7*-containing epitheliocytes in the epithelium (r_s_ = 0.457, *p* = 0.028), between the number of *PAX9*-positive connective tissue cells and the number of *PAX7*-containing connective tissue cells (r_s_ = 0.567, *p* = 0.003), between the number of *PAX9*-containing connective tissue cells and the number of *RYK*-containing connective tissue cells (r_s_ = 0.524, *p* = 0.006), between the number of *PAX7*-containing epitheliocytes in the epithelium and the number of *PAX7*-containing connective tissue cells (r_s_ = 0.574, *p* = 0.004), between the number of *PAX7*-containing connective tissue cells and the number of *RYK*-containing epitheliocytes in the epithelium (r_s_ = 0.549, *p* = 0.006), between the number of *PAX7*-containing connective tissue cells and the number of *RYK*-containing connective tissue cells (r_s_ = 0.439, *p* = 0.025). The correlations between the factors in isolated cleft palate affected tissue are summarized in Table 4.

## 4. Discussion

The formation of non-syndromic orofacial clefts is still unclear and there is a limited amount of information about the differences in genetic factors and signaling pathways in specific types of craniofacial clefts, such as in case of unilateral or bilateral cleft lip, and isolated cleft palate. Our study showed that there are statistically significant differences for the number of *PAX7*, *PAX9*, and *RYK*-positive structures between the control group tissue and different types of cleft affected tissue.

Our research showed that there are statistically significant differences for the number of *PAX7*-positive cells in the epithelium and connective tissue between the control group and the unilateral but not bilateral and isolated cleft palate affected tissue. *PAX7* is a transcription factor involved within the process of craniofacial region development and regulates the formation and differentiation of neural crest cells that form the connective tissue of the orofacial region [21]. Genome-wide association studies have shown that mutations in the *PAX7* gene are associated with the formation of craniofacial clefts [22,23,24]. Our study suggests that *PAX7* could be more functionally involved with the development of specific types of craniofacial clefts such as unilateral cleft lip, and less involved within the formation of bilateral cleft lip and isolated cleft palate, however, further research could help to elaborate this possible functional and pathogenetic connection of *PAX7* with specific cleft types.

The data notified statistically significant differences in the number of *PAX9*-containing structures in the connective tissue only between the control and the unilateral cleft lip group, while such difference was notified between the control group and bilateral cleft lip affected tissue only within the epithelium. The comparison of the control group and isolated cleft palate group showed statistically significant differences in the number of *PAX9*-containing cells both within the epithelium and within the connective tissue. *PAX9* is a transcription factor that has been previously described as necessary for the regulation of palatogenesis [25]. Dysfunction of *PAX9* has been previously associated with the development of craniofacial abnormalities, such as cleft palate and tooth agenesis [26,27]. Mice models have shown that *PAX9* gene deletion and downregulation of *PAX9* causes the formation of cleft secondary palate [28] and associations have been found with *PAX9* and the development of cleft lip in mice [29]. *PAX9* has been linked with the formation of cleft lip with or without cleft palate in humans [30]. Our study results suggest that the number of *PAX9*-positive structures could be different in different cleft affected tissue depending on the cleft type which could affect the pathogenetic pathways in each cleft type. The significant immunoreactivity of *PAX9* in the surface epitheliocytes and connective tissue cells of isolated cleft palate seems to emphasize the interaction between the epithelium and the underlying connective tissue in this specific type of cleft, which could affect tissue growth and remodeling during cleft formation, while the significant presence of *PAX9* within the unilateral and bilateral cleft lip affected tissue and possible involvement in cleft pathogenesis within these specific types of clefts could not be excluded.

Statistically significant differences were found in the number of *RYK*-positive cells and structures in the epithelium and connective tissue between the controls and all three cleft types analyzed in this study. The available information about *RYK* function and formation of clefts is quite limited, but the association between orofacial cleft formation and *RYK* dysfunction and the loss of activity of *RYK* has been previously found in humans [14]. Our study results could suggest that the significant presence of *RYK*-positive cells compared to controls in different cleft types might be explained by the possible similarities of the pathogenetic signaling mechanisms of cleft formation where *RYK* involvement is present.

Multiple statistically significant correlations were found between *PAX7*, *PAX9*, and *RYK* within the cleft affected tissue groups which most likely could be explained by the interaction between these factors in the developing orofacial region during the postnatal period.

An interesting question relates to the intercorrelation of these specific gene proteins. A statistically significant strong correlation was detected between *PAX9*-containing epitheliocytes in the surface epithelium and *PAX9*-containing connective tissue cells in the tissue of unilateral cleft lip. Similarly, a strong correlation was also found between *PAX9*-positive structures within the epithelium and *PAX9*-positive structures in the connective tissue in the isolated cleft palate affected tissue. A very strong correlation between *PAX9*-positive structures in the epithelium and *PAX9*-positive connective tissue cells was found in bilateral cleft lip affected tissue which might indicate a stronger interaction between the cleft affected epithelium and the underlying connective tissue when compared to other types of clefts. Previous studies have concluded that *PAX9* has an involvement not only in the formation of the palatal region but also the upper lip region [29]. These correlations of *PAX9*-positive structures between connective tissue and the oral cavity epithelium in all types of cleft affected tissue may indicate an interaction and the presence of possibly similar pathogenetic mechanisms within the different cleft types analyzed in this study.

Multiple statistically significant moderate correlations were found between *PAX7*, *PAX9*, and *RYK* within the unilateral cleft lip and isolated cleft palate affected tissue. Both *PAX7* and *PAX9* are involved with craniofacial region development [31]. The correlations between *PAX7* and *PAX9* could be explained by their interaction within Wnt and Notch signaling pathways which play an important regulatory role during the formation of orofacial structures [8,32]. *RYK* also functions within the Wnt signaling pathway which also interacts with *PAX9*. Wnt signaling can interact with and activate Fgf (fibroblast growth factor) signaling and modulates *PAX9* to provide repression of DKK (Dickkopf) protein, an inhibitor of the canonical Wnt signaling pathway, causing a positive feedback loop within the craniofacial development process [33]. The disruption within these molecular signaling pathways could eventually lead to the formation of craniofacial clefts. These interactions could explain the correlations between the factors within the different cleft affected tissue.

A very strong statistically significant correlation was notified between *PAX7*-containing epitheliocytes in the surface epithelium and *RYK*-containing epitheliocytes in the surface epithelium within bilateral cleft lip affected tissue. Similarly, a strong correlation between *PAX7*-positive epitheliocytes in the epithelium and *RYK*-positive structures in the epithelium was found in isolated cleft palate affected tissue, but in unilateral cleft lip affected tissue this correlation was only moderate. This possible interaction between *PAX7* and *RYK* is most likely indirect within different Wnt signaling pathways which regulate tissue remodeling and growth processes within the developing orofacial region [34,35]. The differences in the strength of the correlation may indicate differences in factor interaction, pathogenic signaling, and development within the different types of cleft affected tissue.

A limitation of this study is the use of only immunohistochemistry to detect the presence of *PAX7*, *PAX9*, and *RYK* in cleft affected tissue—using additional methods such as gene amplification and in situ hybridization would provide a good addition to this study. The use of these additional techniques is planned for future work. Another limitation is the size of the control group which was relatively small and in which the collection of tissue material is complicated due to ethical concerns.

## 5. Conclusions

1. Transcription factors *PAX7* and *PAX9* are variably involved in the postnatal morphopathogenesis of different facial cleft types: *PAX7* is stably associated with the formation of unilateral cleft lip, while *PAX9* relates more towards the isolated cleft palate.
2. The participation of cleft candidate gene *RYK* in all patterns of facial clefting is proven by its stable appearance in all cleft-affected tissue postnatally.
3. Interactions between *PAX7*, *PAX9*, and *RYK* prove the involvement of all of these factors in the clefting process via the possibly similar signaling pathways disrupted by other still unknown factor/s that influence the gene expression during postnatal life.

## Figures and Tables

**Table 1:** Summary of semiquantitative evaluation showing the median values of *PAX7*, *PAX9*, and *RYK* immunoreactivity in the control group, unilateral cleft lip patient group, bilateral cleft lip patient group, and isolated cleft palate patient group.

	*PAX7*		*PAX9*		*RYK*	
	E	CT	E	CT	E	CT
Controls	+/++	+/++	++	0	0/+	+
Unilateral cleft lip	+/++	+++	+/++	+	++/+++	++/+++
Bilateral cleft lip	++	+/++	+	0/+	++/+++	++/+++
Isolated cleft palate	+/++	++/+++	0/+	+	++/+++	++/+++
H	25.804	28.955	28.308	33.917	22.868	18.307
*p*	<0.001	<0.001	<0.001	<0.001	<0.001	<0.001

Abbreviations: *PAX7*—Paired box 7; *PAX9*—Paired box 9; *RYK*—Receptor-like tyrosine kinase; E—epithelium; CT—connective tissue; H—Kruskal–Wallis H test statistic; *p*—*p*-value; 0—no immunopositive structures; 0/+—a rare occurrence of immunopositive structures; +—a few immunopositive structures; +/++—a few to moderate number of immunopositive structures; ++—a moderate number of immunopositive structures; ++/+++—a moderate to numerous immunopositive structures; +++—numerous immunopositive structures.

**Table 2:** Correlations between paired box 7 (*PAX7*), paired box 9 (*PAX9*), and receptor-like tyrosine kinase (*RYK*) immunopositive structures in unilateral cleft lip affected tissue based on Spearman’s rank correlation coefficient calculation (r_s_—Spearman’s rho value).

Strength of Correlation	Correlations Between *PAX7*, *PAX9*, and *RYK*-Containing Structures in Unilateral Cleft Lip Affected Epithelium and Connective Tissue	r_s_	*p*-Value
Strong: 0.6–0.8	*PAX9* in epithelium and *PAX9* in connective tissue	0.618	<0.001
Moderate: 0.4–0.59	*PAX7* in epithelium and *PAX7* in connective tissue	0.572	<0.001
	*RYK* in epithelium and *RYK* in connective tissue	0.562	<0.001
	*PAX7* in connective tissue and *PAX9* in connective tissue	0.531	0.001
	*RYK* in epithelium and *PAX9* in connective tissue	0.430	0.009
	*RYK* in epithelium and *PAX7* in epithelium	0.407	0.014
	*RYK* in epithelium and *PAX9* in epithelium	0.400	0.016
Weak: 0.2–0.39	*RYK* in connective tissue and *PAX9* in connective tissue	0.354	0.034

**Table 3:** Correlations between paired box 7 (*PAX7*), paired box 9 (*PAX9*), and receptor-like tyrosine kinase (*RYK*) immunopositive structures in bilateral cleft lip affected tissue based on Spearman’s rank correlation coefficient calculation (r_s_—Spearman’s rho value).

Strength of Correlation	Correlations Between *PAX7*, *PAX9*, and *RYK*-Containing Structures in Bilateral Cleft Lip Affected Epithelium and Connective Tissue	r_s_	*p*-Value
Very strong: 0.8–1.0	*PAX9* in epithelium and *PAX9* in connective tissue	0.882	<0.001
	*PAX7* in epithelium and *RYK* in epithelium	0.869	<0.001

**Table 4:** Correlations between paired box 7 (*PAX7*), paired box 9 (*PAX9*), and receptor-like tyrosine kinase (*RYK*) immunopositive structures in isolated cleft palate affected tissue based on Spearman’s rank correlation coefficient calculation (r_s_—Spearman’s rho value).

Strength of Correlation	Correlations Between *PAX7*, *PAX9*, and *RYK*-Containing Structures in Isolated Cleft Palate Affected Epithelium and Connective tissue	r_s_	*p*-Value
Strong: 0.6–0.8	*PAX7* in epithelium and *RYK* in epithelium	0.685	<0.001
	*PAX9* in epithelium and *PAX9* in connective tissue	0.674	<0.001
Moderate: 0.4–0.59	*PAX7* in epithelium and *PAX7* in connective tissue	0.574	0.004
	*PAX9* in connective tissue and *PAX7* in connective tissue	0.567	0.003
	*PAX9* in epithelium and *PAX7* in connective tissue	0.563	0.003
	*PAX7* in connective tissue and *RYK* in epithelium	0.549	0.006
	*PAX9* in epithelium and *PAX7* in connective tissue	0.549	0.007
	*PAX9* in connective tissue and *RYK* in connective tissue	0.524	0.006
	*PAX9* in epithelium and *RYK* in epithelium	0.524	0.010
	*PAX9* in connective tissue and *PAX7* in epithelium	0.457	0.028
	*PAX7* in connective tissue and *RYK* in connective tissue	0.439	0.025

## Data Availability

The data described and analyzed in this study is available on the request from the corresponding author. Due to ethical considerations and the use of children tissue material the data is not publicly available.
